# *Gentiana lutea* root aqueous extract mitigates hydroxyurea-induced genotoxicity through antioxidative action and DNA repair: an *in vitro* study in healthy human peripheral blood mononuclear cells

**DOI:** 10.2478/aiht-2025-76-4033

**Published:** 2025-12-30

**Authors:** Ksenija Radošević, Mila Kostić, Marijana Janić, Ivan Jovanović, Maja Živković, Ana Valenta Šobot, Jelena Filipović Tričković

**Affiliations:** University of Belgrade, Vinča Institute of Nuclear Sciences, Belgrade, Serbia

**Keywords:** chemotherapeutics, gene expression, genoprotection, radical scavenging, yellow gentian, ekspresija gena, genoprotekcija, kemoterapeutik, neutralizacija slobodnih radikala, žuta lincura

## Abstract

Hydroxyurea is a chemotherapeutic agent used to treat various conditions, including sickle cell anaemia and myeloproliferative malignancies. However, it has adverse genotoxic effects on normal cells. This *in vitro* study aimed to explore the genoprotective potential of yellow gentian (*Gentiana lutea* L.) root extract (GRE) against hydroxyurea-induced DNA damage in primary human peripheral blood mononuclear cells. We measured total phenolic and flavonoid GRE content (TPC and TFC, respectively) and its capacity to scavenge free radicals using the DPPH and ABTS assays. Before exposure to hydroxyurea, mononuclear cells were treated with non-cytotoxic and non-genotoxic GRE concentrations to assess their genoprotective (CBMN assay) and antioxidative effects (PAB and GSH assays). We also wanted to see how they affected the expression of DNA repair genes *PARP1*, *OGG1*, and *MnSOD*. GRE TPC was 8.42 mg GAE/g while the TFC was below the detection limit. Even so, GRE displayed radical-scavenging activity and restored hydroxyurea-disrupted cellular redox homeostasis, as PAB values returned to normal and GSH levels rose. GRE pre-treatment significantly reduced hydroxyurea-induced DNA damage in a concentration-dependent manner. *PARP1* and *MnSOD* were upregulated, but not *OGG1*, which indicates GRE’s selective action. Our findings confirm its genoprotective effects against hydroxyurea-induced DNA damage in peripheral blood mononuclear cells, indicate a complex mechanism of action, and call for further research of this promising compound against secondary genotoxic effects of hydroxyurea.

## Abstract (hr)

Hidroksiureja je kemoterapijski lijek koji se koristi za liječenje različitih stanja, uključujući anemiju srpastih stanica i mijeloproliferativne malignitete. Štetni učinci hidroksiureje očituju se kao sekundarni genotoksični učinci na normalne stanice, što naglašava potrebu za istraživanjem prirodnih genoprotektivnih sredstava koja bi mogla pružiti zaštitu normalnim stanicama. Cilj ovoga istraživanja bio je ispitati potencijal ekstrakta korijena *Gentiana lutea* L. u sprječavanju oštećenja DNA izazvanog hidroksiurejom u primarnim ljudskim mononuklearnim stanicama periferne krvi *in vitro*. Ukupni sadržaj fenola i flavonoida u ekstraktu (TPC i TFC) određen je, a sposobnost ekstrakta da neutralizira slobodne radikale procijenjena je pomoću DPPH i ABTS testa. Prije izlaganja hidroksiureji, mononuklearne stanice izložene su necitotoksičnim i negenotoksičnim koncentracijama ekstrakta za procjenu genoprotektivnih (CBMN test), antioksidacijskih (PAB i GSH testovi) te utjecaja na ekspresiju gena uključenih u popravak DNA i antioksidacijsku zaštitu (*PARP1*, *OGG1* i *MnSOD*). TPC ekstrakta iznosio je 8,42 mg GAE/g, a TFC bio je ispod granice detekcije. Ekstrakt je pokazao sposobnost uklanjanja slobodnih radikala te je obnovio narušenu staničnu redoks homeostazu izazvanu hidroksiurejom, što se očitovalo normalizacijom PAB vrijednosti i povećanjem razine GSH. Predtretman s ekstraktom značajno je smanjio oštećenja DNA izazvana hidroksiurejom, i to na način ovisan o koncentraciji. Povećana ekspresija *PARP1* i *MnSOD* gena, ali ne i *OGG1*, sugerira selektivan učinak ekstrakta na aktivaciju gena uključenih u popravak oštećenja DNA i antioksidacijsku zaštitu. Ovi nalazi pokazuju da ekstrakt djeluje genoprotektivno protiv oštećenja DNA izazvanog hidroksiurejom u mononuklearnim stanicama periferne krvi putem složenog mehanizma, što upućuje na njegov mogući potencijal u prevenciji sekundarnih genotoksičnih učinaka hidroksiureje.

Hydroxyurea (hydroxycarbamide, HU) is a chemotherapeutic agent with a chemical structure analogous to urea (CH_4_N_2_O_2_). It is used to treat various myeloproliferative malignancies, including chronic myeloid leukaemia and sickle cell anaemia (1). Its short-term adverse effects are seen as reversible toxicities on growth and development. However, one long-term study of HU treatment (2) reported late adverse effects such as reversible leucopoenia and myelosuppression, drug-induced dermopathy with characteristics of dermatomyositis, and infertility. As an antimetabolite, HU exerts antiproliferative effects by inhibiting ribonucleotide diphosphate reductase (RDR) and depleting the dNTP pool, which ultimately results in cell cycle arrest in the S-phase (1). However, HU also induces secondary genotoxic effects in normal, healthy cells, especially when applied at higher doses or for long, and/or when the S-phase checkpoint mechanisms are ineffective (3, 4), so the cell continues DNA synthesis despite the lack of dNTPs, making single-stranded DNA unprotected and prone to single-strand breaks (SSB). Further progression of the cell cycle leads to the fork collapse and the subsequent formation of double-strand breaks (DSB).

On the other hand, the effects of HU on oxidative stress parameters remain ambiguous. Some studies report its antioxidant effect through radical scavenging and elevated expression of antioxidant genes (5), while other studies claim that it induces reactive oxygen species (ROS) formation and, indirectly, genotoxicity (1, 6). Its hydroxyl amine group initiates a chain reaction of free radicals, which results in the generation of hydroxyl radicals that directly react with DNA bases and mostly generate 8-oxodeoxyguanosine (8-oxo-dG) (7). Additionally, HU alters RNA synthesis and transcription in a bimodal, dose-dependent manner, suggesting enzyme-mediated cytotoxic and genotoxic properties (8).

One of the promising natural agents that could counteract HU genotoxicity is the *Gentiana lutea* root extract (GRE), as it has already shown promising genoprotective effects against various xenobiotics (9, 10). *G. lutea* L. is a medicinal herb from the *Gentianaceae* family, traditionally used to relieve gastrointestinal and cardiovascular disorders, as well as an antidiabetic and antirheumatic remedy (11, 12). *Gentiana* species contain iridoids, triterpenoids, flavonoids, xanthones, and other secondary metabolites. Molecules in secondary metabolites differ in hydroxy, methoxy, glycoside, or complex substituents and form more than 590 compounds (13). Such complex chemical composition can have diverse biological properties. Our previous studies show that the genoprotective effect of its phytoconstituents are mediated by antioxidative activity (9, 14) and the activation of the DNA repair mechanisms, including homologous recombination (15) and, arguably, base excision repair (16). Its antioxidative effect relies on dual action: direct ROS scavenging and the activation of nuclear factor erythroid 2-related factor 2 (Nrf2), which stimulates the expression of antioxidant defence enzymes (9). However, at high concentrations or prolonged treatment, GRE or secoiridoid glycosides isolated from it can have a cytotoxic effect on peripheral blood mononuclear cells (PBMC) by activating apoptosis and necroptosis (16).

Given the antioxidant capacity of GRE and its role in activating DNA repair mechanisms, and taking into account that the genotoxic effect of HU is mediated, among other pathways, by ROS generation, we hypothesised that GRE pre-treatment could protect against HU-induced genotoxic damage on the cellular level.

## MATERIALS AND METHODS

To test this hypothesis, before we exposed PBMCs to HU, we pre-treated them with a range of non-cytotoxic, non-genotoxic GRE concentrations to assess their genoprotective and antioxidative effects, as well as their impact on the expression of the following genes: *MnSOD*, encoding manganese superoxide dismutase involved in antioxidation, *OGG1*, encoding 8-oxoguanine DNA glycosylase, and *PARP 1*, encoding poly(ADP-ribose) polymerase 1, both involved in DNA repair.

The study was conducted in accordance with the Declaration of Helsinki and approved by the Ethics Committee of the Vinča Institute of Nuclear Sciences, Serbia (approval No. 116-5-2/2023-000).

Commercially available root of *G. lutea* was purchased from the Dr Josif Pančić Institute for the Research of Medicinal Plants, Belgrade, Serbia. For the isolation of mononuclear cells we used the lymphocyte separation medium A (LSM-A) and for cultivation of non-adherent cells the Roswell Park Memorial Institute medium 1640 (RPMI 1640), and phytohaemagglutinin (PHA-M), all produced by Capricorn Scientific (Ebsdorfergrund, Germany). Chemical substances used in the analysis of total phenolic and total flavonoid content and measurement of oxidative stress parameters included horseradish peroxidase (HRP), 3,3′,5,5′-tetramethylbenzidine (TMB), chloramine T, hydrogen peroxide, uric acid, 1-methyl-2-phenylindole, tris base, acetonitrile, gallic acid, quercetin, 2,2′-diphenyl-1-picrylhydrazyl (DPPH), 2,2′-azino-bis(3-ethylbenzothiazoline-6-sulfonic acid) diammonium salt (ABTS), Triton X-100, 1,1,3,3-tetramethoxypropane, methanesulphonic acid, and acetaldehyde. For protein concentration determination we used bovine serum albumin (BSA) and the Folin-Ciocalteu reagent. For the cytokinesis block micronucleus (CBMN) assay we used cytochalasin B and Giemsa dye. All these substances and reagents are the products of Sigma Aldrich (St. Louis, MO, USA). For the real-time quantitative polymerase chain reaction (RT-qPCR) we used the TRIzol^™^ reagent and the RevertAid First strand cDNA synthesis kit, both purchased from ThermoFisher Scientific Inc. (Waltham, MA, USA).

### Preparation of the *G. lutea* root extract

GRE was prepared by mincing the *G. lutea* root and pouring boiling distilled water over it in a 1:5 (m/v) ratio, as described in detail elsewhere (17). After cooling, GRE was filtered, frozen at −20 °C, and then lyophilised under vacuum. The lyophilisate was stored in a desiccator at 4 °C. Before use, it was dissolved in deionised water and filtered through Minisart^®^ filters with a pore diameter of 0.2 µm (Sartorius, Göttingen, Germany). The GRE TPC and TFC content was determined as described by Singleton et al. (18) and Chang et al. (19), respectively, with minor adjustments made for 96-well microplates. A range of gallic acid and quercetin concentrations (0.625–80 µg/mL) served to construct respective calibration curves for TPC and TFC. GRE concentrations for the determination of TPC were 0.125 mg/mL, 0.25 mg/mL, and 0.5 mg/mL and for TFC 0.5 mg/mL, 2 mg/mL, and 5 mg/mL.

The absorbances were read at 700 nm for TPC and at 415 nm for TFC using a microplate reader (Sunrise, Tecan Group Ltd, Switzerland). The final results are expressed as mg of gallic acid equivalents per g of dry extract (mg GAE/g) for TPC and as mg of quercetin equivalents (QE) per g of dry extract (mg QE/g) for TFC and presented as the mean value of three measurements ± standard deviation.

The capacity of GRE to neutralise the 2,2-diphenyl-1-picrylhydrazyl (DPPH^•^) radical was determined using a spectrophotometric method adapted for 96-well microplates, as described elsewhere (20). The assay is based on monitoring the colour change from purple of the stable DPPH^•^ solution to yellow of its reduced, DPPH-H form. GRE was analysed in a concentration range of 0.02–1.5 mg/mL. The absorbance was measured on a microplate reader (Sunrise) at 517 nm.

Spectrophotometry was also used to determine GRE’s capacity to neutralise the 2,2′-azino-bis(3-ethylbenzothiazoline-6-sulfonic acid (ABTS^•+^) radical cation, as described by Re et al. (21). The assay is based on ABTS^•+^ reduction in the presence of hydrogen-donating antioxidants, which results in decolourisation. GRE was analysed in a concentration range of 0.02–1.50 mg/mL. The absorbance was measured on a microplate reader (Sunrise) at 700 nm.

The percentage of inhibition (%) for both assays was calculated according to the following equation:

[1] $$\text{Inhibition}\;\left(\%\right)=\frac{\text{Acontrol}-\text{Asample}}{\text{Acontrol}}\times 100$$

where A_control_ is the absorbance of the control and A_sample_ is the absorbance of the sample. All tests were done in triplicate, and the obtained data were further analysed in Origin Pro, version 8.0 (OriginLab Corporation, Northampton, MA, USA). The results are expressed as half-maximal inhibition (IC_50_) in mg/mL.

### Isolation, cultivation, and treatments of human peripheral blood mononuclear cells

Peripheral blood for PBMC cultures was taken from three healthy volunteers, aged 20–40 years, into Li-heparin vacutainers (Becton Dickinson, Plymouth, UK). PBMCs were isolated in a mononuclear cell isolation medium (Capricorn Scientific) and resuspended in an RPMI 1640 medium supplemented with 1 % penicillin-streptomycin solution, 3 % PHA-M (Capricorn Scientific), and 10 % foetal bovine serum (FBS) (complete medium) at a concentration of 1×10^6^ cells/mL.

After cultivation for 72 h, the cell cultures were first treated with different concentrations of HU (50, 100, and 200 µmol/L) for 24 h to find the concentration that induces genotoxic effects without significantly inhibiting cell proliferation. To find the GRE concentration that does not induce geno- or cytotoxic effects, the cell cultures were serially treated with 0.25, 0.5, 1, and 2 mg/mL of GRE for 24 h.

Having determined the optimal concentrations of both compounds, we continued as follows: the cells were pre-treated with a range of non-cytotoxic and non-genotoxic GRE concentrations for 24 h and then exposed to 100 µmol/L of HU (which induces significant genotoxic effect but does not affect proliferation) for additional 24 h. The cells were cultured at 37 °C, in a humidified atmosphere with 5 % CO_2_ (CO_2_ incubator, ESCO, Singapore).

The genoprotective effect of GRE against HU was evaluated with the CBMN assay and by monitoring micronucleus frequency and proliferation indices.

We also determined the pro-oxidant/anti-oxidant balance (PAB), GSH levels, and the expression of genes involved in DNA damage repair (*OGG1* and *PARP1*) and antioxidant repair (*MnSOD*) to identify mechanisms by which GRE accomplishes genoprotective effect. Untreated cell cultures served as negative, and HU-alone-treated cultures as positive control.

### Cytokinesis-block micronucleus assay

The CBMN assay was carried out as described by Fenech (22) and served to optimise HU and GRE concentrations and to evaluate the genoprotective potential of GRE pre-treatments. Whole blood aliquots (0.5 mL) were grown in the complete medium, and cytochalasin B added 44 h later at the final concentration of 4 µg/mL. Cells were harvested and prepared slides stained with 10 % Giemsa solution. At least 1000 binuclear cells were analysed under the AxioImager A1 epifluorescence microscope (Carl Zeiss, Jena, Germany) at 400× and 1000× magnification, following the HUman MicroNucleus (HUMN) Project scoring criteria (23).

The cytokinesis-block proliferation index (CBPI) was determined using the following equation:

[2] $$\text{CBPI}=\frac{\left[\text{MI}+2\text{MII}+3\left(\text{MIII}+\text{MIV}\right)\right]}{\text{N}}$$

where MI–IV denotes the number of cells with one to four nuclei and N denotes the total number of cells. The CBPI proliferation threshold was set to 80 %, which means that further experiments used only the concentrations that did not reduce cell proliferation below 80 %.

### Pro-oxidant/antioxidant balance analysis

The oxidative stress effects of HU and the mitigating capacity of GRE were assessed with the PAB in PBMCs lysates as described by Alamdari et al. (24). Optical density (OD) was measured on the Sunrise absorbance microplate reader at 450 nm. PAB results are expressed as the percentage of untreated control (100 %).

### Determination of the reduced glutathione levels

GSH levels were determined following the Ellman’s method (25). This assay is based on the reduction of 5,5-dithio-bisnitrobenzoic acid (DTNB) by GSH to a coloured 2-nitro-5-mercaptobenzoic acid. The optical density (OD) was read on the Sunrise absorbance microplate reader at 412 nm and GSH levels calculated as µmol/mg of total proteins in the cell lysate from the standard curve obtained from a series of solutions with known GSH concentrations in the range 0.00–1.25 mmol/L. The final concentration is presented as the percentage of untreated control (100 %).

### Gene expression analysis

The relative expression of *OGG1*, *PARP1*, and *MnSOD* was determined with the RT-qPCR. Total RNA was extracted from treated PBMC cultures using the TRIzol reagent and reverse-transcribed using the RevertAid First strand cDNA synthesis kit with oligo-dT18 according to manufacturer’s (ThermoFisher Scientific) instructions (26). Expression levels were measured on an Applied Biosystems Real-Time 7500 system (Applied Biosystems, Inc., Foster City, CA, USA) using the following TaqMan^®^ primers: Hs00242302_m1 for *PARP1*, Hs00213454_m1 for *OGG1*, and Hs00167309_m1 for *MnSOD*. For internal control we used the relative expression of the mRNA peptidylprolyl isomerase A gene (*PPIA)* (Hs99999904_m1).

All reactions were done in duplicate in a 96-well plate, as follows: initial cycle at 50 °C for 2 min, followed by one cycle at 95 °C for 10 min, 40 cycles at 95 °C for 15 s, and the final cycle at 60 °C for 1 min. Relative expression of the target genes was analysed using the comparative CT method (2^−ΔΔCt^ method) as described elsewhere (27).

### Statistical analysis

All the experiments were performed on three independent samples and repeated twice. The results are presented as means ± standard errors of the mean (mean±SE). The results were analysed with the SPSS software for Windows 10, version 20 (IBM, Armonk, NY, USA). The normality of data distribution was assessed with the Shapiro-Wilk test due to the small sample size. The test indicated no significant deviation from normal distribution, supporting the use of a parametric test for statistical analysis. Therefore, differences between groups were evaluated using the one-way ANOVA, with significance set at P<0.05.

## RESULTS

### GRE total phenolic and flavonoid content and radical neutralisation capacity

The TPC of GRE was 8.42±0.33 mg GAE/g, while TFC was below the detection even at the highest tested concentration.

**Figure 1:**
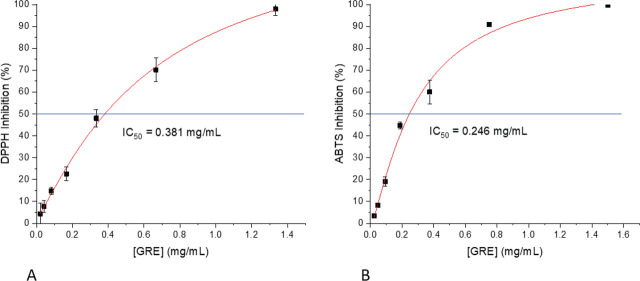
Radical-scavenging capacity of GRE determined with the DPPH (A) and ABTS assays (B), expressed as a percentage of inhibition. GRE – yellow gentian (*Gentiana lutea* L.) root extract; DPPH – 2,2-diphenyl-1-picrylhydrazyl; ABTS – 2,2′-azino-bis(3-ethylbenzothiazoline-6-sulfonic acid

Figure 1 shows the radical-scavenging capacity of GRE, which increased with concentrations. The calculated for DPPH^•^ IC_50_ was 0.371±0.031 mg/mL, while the maximum inhibition of 97.9 % was achieved with 1.33 mg/mL. The ABTS^•^ IC_50_ was 0.246±0.018 mg/mL, and the maximum inhibition of 99.6 % was achieved with 1.5 mg/mL.

### Genotoxicity and cell proliferation effects of hydroxyurea and gentian root extract

**Figure 2:**
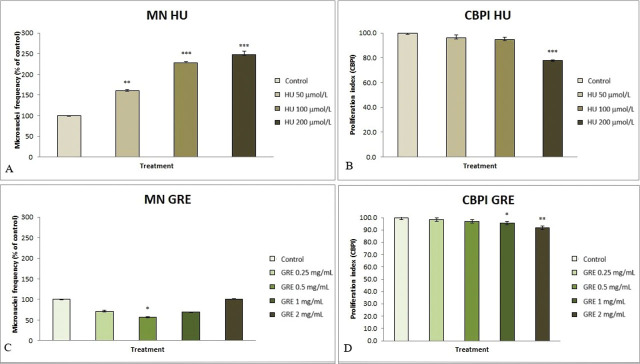
Genotoxicity (MN frequency, A and C panels) and cytotoxicity (CBPI, B and D panels) in PBMC cultures treated with GRE or HU alone. ^*^P<0.05, ^**^P<0.01, ^***^P<0.001 treatments vs control. CBPI – cytokinesis-block proliferation index; GRE – yellow gentian (*Gentiana lutea* L.) root extract; HU – hydroxyurea; MN – micronucleus; PBMC – peripheral blood mononuclear cell

**Figure 3:**
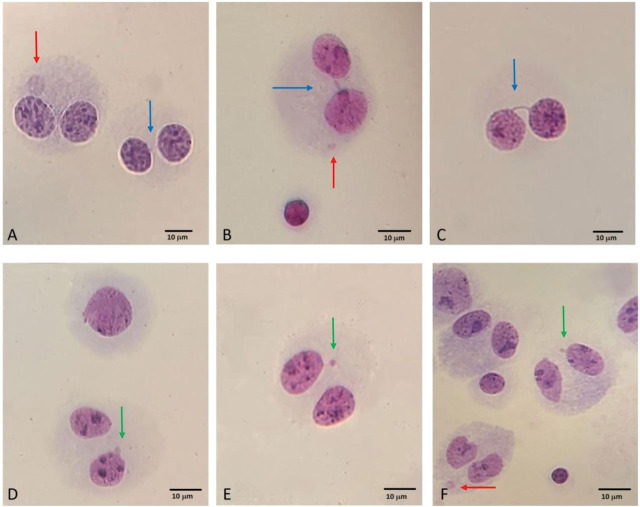
Representative photomicrographs of binuclear cells (CBMN assay): HU treatment (A, B, C), micronuclei (red arrows) and nucleoplasmic bridges (blue arrows); GRE pre-treatment and HU treatment (D, E, F), micronuclei (red arrows) and nuclear buds (green arrows) (scale bar=10 µm). CBMN – cytokinesis-block micronucleus; GRE – yellow gentian (*Gentiana lutea* L.) root extract; HU – hydroxyurea

Figure 2A and B and Table 1 show that HU treatment increased MN frequency and lowered proliferation of PBMCs in a concentration-dependent manner. Compared to control, the MN frequency increased significantly with all HU concentrations, but cell proliferation remained relatively normal until the highest concentration of 200 µmol/L was applied, which significantly reduced the proliferation index to 77 % (P<0.001). Interestingly, nucleoplasmic bridges were observed with all HU concentrations, indicating its clastogenic potential (Figure 3, upper panels; Table 1). Considering that 100 µmol/L of HU induced a significant genotoxic effect without affecting cell proliferation, we selected this concentration for further experiments.

**Table 1:** Frequencies of micronuclei, nuclear buds, and nucleoplasmic bridges in individual treatments (optimisation experiment) and PBMC cultures pre-treated with GRE followed by HU treatment

	**Micronucleus frequency (per 1000 binuclear cells)**	**Nuclear bud frequency (per 1000 binuclear cells)**	**Nucleoplasmic bridge frequency (per 1000 binuclear cells)**
**Optimisation experiment**			
Control	8.17±0.4	6.33±0.49	/
HU 50 µmol/L	13.07±0.4	12±0.89	1.66±0.31
HU 100 µmol/L	18.67±0.33	17.83±0.95	3.17±0.31
HU 200 µmol/L	20.25±0.63	18.67±1.28	4.67±0.49
GRE 0.25 mg/mL	5.75±0.44	8.83±0.6	/
GRE 0.5 mg/mL	4.67±0.54	8.5±0.76	/
GRE 1 mg/mL	5.67±0.4	9.5±0.92	/
GRE 2 mg/mL	8.25±0.6	10.83±0.83	/
**Pre-treatment (100 µmol/L HU+GRE)**			
Control	7.83±0.7	5.5±0.43	/
HU 100 µmol/L	16.83±0.48	16.17±0.87	/
GRE 0.25 mg/mL+HU	11.92±1.19	25.67±0.84	/
GRE 0.5 mg/mL+HU	10.58±0.8	25.17±0.65	/
GRE 1 mg/mL+HU	11.33±0.67	35.67±1.98	/
GRE 2 mg/mL+HU	9.25±0.93	30.17±1.2	/

GRE – yellow gentian (*Gentiana lutea* L.) root extract; HU – hydroxyurea, PBMC – peripheral blood mononuclear cell

In contrast to HU, GRE reduced MN frequency significantly only at the concentration of 0.5 mg/mL (Figure 2C; Table 1) and cell proliferation at the concentrations of 1 and 2 mg/mL (95.5 % and 89.88 %, P<0.05 and P<0.01, respectively) (Figure 2D). Since all GRE concentrations lowered MN frequency without lowering CBPI below the 80 % threshold, they were all selected for further genoprotective evaluation against HU.

### Genoprotective potential of GRE

**Figure 4:**
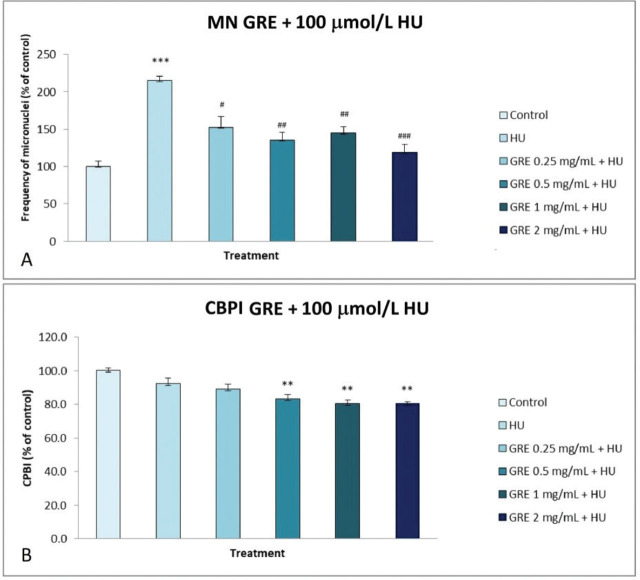
Genoprotective potential of GRE (CBMN assay): MN frequency (A) and CBPI (B) in PBMC cultures pre-treated with GRE followed by HU treatment. ^#^P<0.05; ^##^P<0.01; ^###^P<0.001 – GRE pre-treatment vs HU treatment; ^***^P<0.001 – HU treatment vs control. CBPI – cytokinesis-block proliferation index; GRE – yellow gentian (*Gentiana lutea* L.) root extract; HU – hydroxyurea; MN – micronucleus; PBMC – peripheral blood mononuclear cell

Figure 4 shows that pre-treatment with all GRE concentrations significantly countered the HU-induced MN frequency increase in a concentration-dependent manner, approaching control values with the highest concentration (118 %, P<0.001, Figure 4A). Higher frequency of nuclear buds was observed in all cultures pre-treated with GRE (Figure 3, lower panels; Table 1). Cultures pre-treated with a GRE concentrations of 0.5, 1, and 2 mg/mL had a significant drop in CBPI compared to control, but none compared to HU treatment alone (Figure 4B).

### PAB and GSH

**Figure 5:**
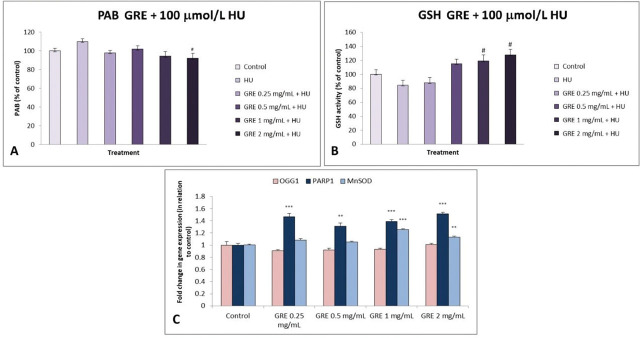
Oxidative stress parameters (A and B panels) and gene expression (C panel) in PBMC in cultures exposed to GRE and HU. #P<0.05 – GRE pre-treatment vs HU treatment; ^**^P<0.01 and ^***^P<0.001 – GRE pre-treatment vs control. GRE – yellow gentian (*Gentiana lutea* L.) root extract; GSH – reduced glutathione; HU – hydroxyurea; PAB – pro-oxidant/antioxidant balance; PBMC – peripheral blood mononuclear cell

Figure 5 shows that 100 µmol/L of HU increased PAB values by approximately 10 % compared to control, but the change was not significant. GRE pre-treatment lowered PAB to near control values, but the drop was significant (P<0.05) compared to HU-alone treatment only with the highest GRE concentration of 2 mg/mL (92.13 %).

Comparable to PAB results, HU lowered the GSH levels by almost 20 %, but the difference from control was not significant. GRE pre-treatment led to a concentration-dependent increase in GSH activity, which was significantly higher compared to HU-alone treatment at the two highest concentrations of 1 and 2 mg/mL (119.57 % and 127.68 %, respectively; P<0.05).

### Changes in DNA-repair and antioxidant gene expression

*OGG1* expression did not change significantly compared to control, regardless of GRE pre-treatment concentrations, whereas *PARP1* expression increased significantly with all of them, reaching the highest, 1.51-fold increase with 2 mg/mL (P<0.001). *MnSOD* expression increased significantly with the two highest concentrations of 1 mg/mL (1.26-fold higher; P<0.001) and 2 mg/mL (1.13-fold higher; P<0.01).

## DISCUSSION

Our findings confirm our hypothesis that pre-treatment with GRE would counter HU-induced genotoxic damage in PBMCs, which was evident at all tested GRE concentrations. The greatest reduction in MN frequency was achieved with the highest concentration of 2 mg/mL. Genoprotective effects of *G. lutea* have previously been reported against food borne mutagens, UV, and ionising radiation and attributed to the radical-scavenging activity and regulation of antioxidant and DNA repair genes (9, 10, 28). However, to the best of our knowledge, our findings are the first to report the protective properties of GRE against HU-induced DNA damage. They also seem to confirm that its genoprotective action is mediated by radical scavenging (29, 30), which mitigates ROS production induced by HU and the depletion in dNTP pool that impairs DNA damage repair (1, 6, 16, 31,32,33,34). Consistent with DPPH and ABTS results obtained in this and other studies (9, 10, 35, 36), GRE pre-treatment lowered the PAB levels and increased GSH in a concentration-dependent manner most likely by upregulating *Nrf2* (9), a transcription factor that regulates the expression of antioxidant genes, including *MnSOD*, and enzymes involved in GSH synthesis and recycling (37, 38) and by upregulating *PARP1*, which has a role in DNA repair (15, 16, 39). GRE pre-treatment did not, however, change the expression of *OGG1,* which encodes the key enzyme in base excision repair (40).

Moreover, the CBMN assay in our study revealed the higher frequency of nuclear buds in all combined GRE+HU treatments. Nuclear buds are protrusions of the nuclear membrane, associated with amplified DNA or nuclear elimination of DNA repair complexes (41). Considering that they seem to be associated with homologous recombination rather than other repair mechanisms (42), our findings suggests that GRE activated homologous recombination in response to HU treatment.

We would like to address the absence of flavonoid content in our aqueous GRE samples, even though it has been reported by some studies (9, 35). This could be owed to aqueous extraction, which is better for polar compounds such as phenolic acids, secoiridoids, and iridoids, while many flavonoids are more soluble in organic solvents (35,36, 43). Even so, the IC_50_ values in this study are similar to those of the ethanolic-aqueous extract reported by Cvetković et al. (9). Aqueous extracts, on the other hand, have a considerable advantage in terms of lower toxicity and better suitability for biomedical applications (44). Furthermore, the antioxidant capacity of GRE may be attributed to non-flavonoid phenolic compounds and other bioactive constituents rather than flavonoids (45).

We would also like to address possible limitations of our study. One concerns control cells, which were not kept in deionised water to mimic the aqueous solution conditions. The other concerns the CBMN assay used, which relied on the older Fenech protocol (22) instead of the more recent one (46) which includes the evaluation of apoptosis and necrosis, as well as the calculation of NDCI in addition to NDI (CBPI). However, our study focused on assessing the potential genoprotective effects of GRE against HU concentrations that are genotoxic but not highly cytotoxic, so evaluating apoptosis and necrosis was not included in the scope of this work.

## CONCLUSION

Regardless of the above limitations, our study clearly confirms the concentration-dependent genoprotective effects of the *G. lutea* root aqueous extract against genomic damage induced by hydroxyurea in peripheral blood mononuclear cells *in vitro*. It also suggests that the mechanism of its genoprotective action is complex and mediated by the extract’s radical-scavenging properties and up-regulation of the *PARP1* and *MnSOD* genes, involved in DNA repair and antioxidant defence, respectively. Although further *in vivo* validation is required, our findings provide a preliminary mechanistic rationale for developing *G. lutea* root extract as a genoprotective agent with potential use for adjuvant therapy, oxidative stress management, and preventive health strategies for patients undergoing long-term treatment with this chemotherapeutic agent.

## Abbreviations

8-oxo-dG: 8-oxo-deoxyguanosine
ABTS^•+^: 2,2′-azinobis-3-ethylbenzothiazoline-6-sulphonic acid radical cation
ARE: antioxidant response elements
BER: base excision repair
BSA: bovine serum albumin
CBMN: cytokinesis-block micronucleus
CBPI: cytokinesis-block proliferation index
dNTP: deoxyribonucleotide
DPPH•: 2,2-diphenyl-1-picryl-hydrazyl radical
DSB: double-strand breaks
DTNB: 5,5-dithio-bis-(2-nitrobenzoic acid)
FBS: foetal bovine serum
GRE: yellow gentian (*Gentiana lutea L., G. lutea*) root extract
GSH: reduced glutathione
HRP: horseradish peroxidase
HU: hydroxyurea
IC_50_: half-maximal inhibitory concentration
LSM: lymphocyte separation medium
MN: micronucleus
Nrf2: nuclear factor erythroid 2-related factor 2
OD: optical density
OGG: oxoguanine glycosylase
PAB: pro-oxidant/antioxidant balance
PBMC: peripheral blood mononuclear cells
PHA-M: phytohaemagglutinin
RDR: ribonucleotide diphosphate reductase
ROS: reactive oxygen species
RPMI 1640: Roswell Park Memorial Institute 1640 medium
RSC: radical scavenging capacity
RT-qPCR: real-time quantitative polymerase chain reaction
SE: standard error of the mean
SSB: single-strand breaks
TB: trypan blue
TFC: total flavonoid content
TMB: 3,3′,5,5′-tetramethylbenzidine
TPC: total phenolic content
